# Poractant alfa versus bovine lipid extract surfactant for infants 24+0 to 31+6 weeks gestational age: A randomized controlled trial

**DOI:** 10.1371/journal.pone.0175922

**Published:** 2017-05-04

**Authors:** Brigitte Lemyre, Christoph Fusch, Georg M. Schmölzer, Nicole Rouvinez Bouali, Deepti Reddy, Nicholas Barrowman, Nicole Huneault-Purney, Thierry Lacaze-Masmonteil

**Affiliations:** 1 Department of Obstetrics, Gynecology and Newborn Care, The Ottawa Hospital, Ottawa, Ontario, Canada; 2 Ottawa Hospital Research Institute, Ottawa, Ontario, Canada; 3 Department of Pediatrics, Children’s Hospital of Eastern Ontario, Ottawa, Ontario, Canada; 4 CHEO Research Institute, Ottawa, Ontario, Canada; 5 Department of Pediatrics, McMaster University, Hamilton, Ontario, Canada; 6 Department of Pediatrics, University of Alberta, Edmonton, Alberta, Canada; 7 Centre for the Studies of Asphyxia and Resuscitation, Neonatal Research Unit, Royal Alexandra Hospital, Edmonton, Alberta, Canada; 8 Department of Respiratory Therapy, The Ottawa Hospital, Ottawa, Ontario, Canada; Hopital Robert Debre, FRANCE

## Abstract

**Objectives:**

To compare the efficacy and safety of poractant alfa and bovine lipid extract surfactant in preterm infants.

**Study design:**

Randomized, partially-blinded, multicenter trial. Infants <32 weeks needing surfactant before 48 hours were randomly assigned to receive poractant alfa or bovine lipid extract surfactant. The primary outcome was being alive and extubated at 48 hours post-randomization. Secondary outcomes included need for re-dosing, duration of respiratory support and oxygen, bronchopulmonary dysplasia, mortality and complications during administration.

**Results:**

Three centers recruited 87 infants (mean 26.7 weeks and 906 grams) at a mean age of 5.9 hours, between March 2013 and December 2015. 21/42 (50%) were alive and extubated at 48 hours in the poractant alfa group vs 26/45 (57.8%) in the bovine lipid extract surfactant group; adjusted OR 0.76 (95% CI 0.30–1.93) (p = 0.56). No differences were observed in the need to re-dose. Duration of oxygen support (41.5 vs 62 days; adjusted OR 1.69 95% CI 1.02–2.80; p = 0.04) was reduced in infants who received poractant alfa. We observed a trend in bronchopulmonary dysplasia among survivors (51.5% vs 72.1%; adjusted OR 0.35 95%CI 0.12–1.04; p = 0.06) favoring poractant alfa. Twelve infants died before discharge, 9 in the poractant alfa group and 3 in the bovine lung extract group. Severe airway obstruction following administration was observed in 0 (poractant alfa) and 5 (bovine lipid extract surfactant) infants (adjusted OR 0.09 95%CI <0.01–1.27; p = 0.07).

**Conclusion:**

No statistically significant difference was observed in the proportion of infants alive and extubated within 48h between the two study groups. Poractant alfa may be more beneficial and associated with fewer complications than bovine lipid extract surfactant. However, we observed a trend towards higher mortality in the poractant alfa group. Larger studies are needed to determine whether observed possible benefits translate in shorter hospital admissions, or other long term benefits and determine whether there is a difference in mortality.

## Introduction

Surfactant replacement therapy is standard of care for preterm infants with respiratory distress syndrome (RDS) and surfactant deficiency. Both randomized controlled trials (RCTs) and meta-analyses have demonstrated improved survival rates and a reduced incidence of pneumothorax in infants who received surfactant. [1–4] Animal derived surfactants have a faster onset of action and are more effective at reducing oxygen needs than non-protein containing synthetic surfactants. [1] Animal-derived surfactants differ in their phospholipids, surfactant proteins B and C and plasmalogens composition and also in their volume of administration; these factors may affect their efficacy and ease of administration. Complications associated with the administration of surfactant, including airway obstruction, bradycardia and desaturation have been reported. [5]

Currently, only bovine extracted surfactants are available in Canada: BLES^®^ (bovine lipid extract surfactant, BLES Biochemicals, London, Ontario) purified from bovine lung lavage and beractant extracted from minced bovine lungs. Most neonatal intensive care units (NICUs) in Canada use bovine lipid extract surfactant, for its observed faster onset of action, shown in one single clinical trial comparing bovine lipid extract surfactant to beractant. [6] The recommended dose is 5 mL/kg, which is the highest for any animal-derived surfactant. A recent prospective cohort study reported severe airway obstruction with marked desaturation and/or bradycardia in 8/39 preterm infants treated with bovine lipid extract surfactant; six of these infants weighed <1000g at birth. [7]

In comparison, Curosurf^®^ (poractant alfa. Chiesi Farmaceutici, Parma, Italy), a surfactant extracted from porcine minced lungs, is most widely used in Europe and the USA. Compared to bovine lipid extract surfactant or beractant, poractant alfa contains a higher concentration of phospholipids and plasmalogens, which is speculated to explain the rapid improvement observed immediately after administration. [8] This has translated, in some studies, into a higher rate of extubation success. [9] A recent meta-analysis compared poractant alfa and beractant in infants with RDS. Treatment with beractant resulted in significantly more death before hospital discharge (Number Needed To Harm (NNTH) of 20, death or bronchopulmonary dysplasia (BPD) (defined as oxygen at 36 weeks) (NNTH 7), patent ductus arteriosus needing treatment (NNTH 4) when compared with an initial dose of 200 mg/kg of poractant alfa. Less re-dosing (needing >1 dose of surfactant) was also reported with poractant alfa. [10]

No study has compared bovine lipid extract surfactant to poractant alfa. The objective of our study was to compare the efficacy of poractant alfa and bovine lipid extract surfactant, at facilitating extubation and reducing pulmonary morbidities and to compare adverse events during surfactant administration in very premature infants. We hypothesized that poractant alfa would increase the proportion of infants alive and extubated within 48 hours after surfactant therapy.

## Materials and methods

Three Canadian university-affiliated tertiary NICUs recruited patients in the trial (NCT01709409). The trial was approved by the Research Ethics Board at each participating hospital: The Ottawa Health Science Network Research Ethics Board (OHSN-REB) (The Ottawa Hospital), the Children's Hospital of Eastern Ontario (CHEO) Research Ethics Committee (CHEO), McMaster Research Ethics Board (McMaster University Children's Hospital) and the University of Alberta Research Ethics Board (Royal Alexandra Hospital). Informed consent was obtained from parents of participating infants. We obtained a Non-Objection Letter from Health Canada (control # 157267) to undertake this trial, as poractant alfa was not available in Canada when the trial was conducted.

### Study population

Eligible infants were those born between 24+0 and 31+6 weeks gestational age (GA), with established RDS requiring intubation and surfactant therapy within their first 48 hours of life. Infants were excluded if they: had life-threatening congenital anomalies or were considered non-viable; were born outside a trial center; required early intubation and ventilation for surgical treatment of a congenital anomaly; were on high-frequency oscillatory ventilation; had anomalies of the upper or lower airway or mandible precluding use of nCPAP or were born after prolonged premature rupture of membranes (<22 weeks GA or >14 days prior to delivery).

Initially, we aimed to recruit infants born between 24+0 and 29+6 weeks GA. Due to slow accrual after 12 months of recruitment (29 patients recruited), the protocol was amended to allow inclusion of infants up to 31+6 weeks gestational age, infants who received high-frequency oscillation ventilation as a primary mode of ventilation (but not those on rescue treatment due to severe ventilation problems pre-surfactant) or those with prolonged premature rupture of membranes up to 27 days. Units had similar criteria regarding the need for intubation and surfactant after birth: Downes’ RDS score [11] >8 or >1 of the following: 1) FiO2 ≥ 40 & rising; 2) pH<7.20 and pCO2>60 (persistent & associated with clinical distress) or 3) recurrent apnea (6/6h requiring stimulation or >2 in 2 consecutive hours or 1 requiring positive pressure inflations). The Downes’ RDS score considers GA, respiratory rate, breath sounds on auscultation, grunting, retractions and fi02 with possible scores ranging from 0 to 12.

Written informed consent was obtained from parents of all infants who participated in the trial. We approached parents of potentially eligible infants both before and after birth.

### Randomization

Patients were randomly assigned to receive poractant alfa (Curosurf^®^, Chiesi Farmaceutici, Parma, Italy), or bovine lipid extract surfactant (BLES^®^ Biochemicals, London, Ontario) (control) in a 1:1 ratio using a computerized random-number generator. Twins or higher multiples were randomized as individual patients. Randomization was stratified by center and by GA category (24–26 weeks and 27–31 weeks), with random block lengths. Allocation was concealed in sealed, opaque, sequentially-numbered envelopes, which were opened by a Respiratory Therapist (RT) not involved in the care of the infant, immediately after intubation.

### Intervention and blinding

Infants received either the study surfactant, poractant alfa or the control surfactant, bovine lipid extract surfactant. The treatment dose for poractant alfa was 2.5 mL/kg (200mg/kg of phospholipids) for the first dose and 1.25 mL/kg (100mg/kg) for repeat doses. The treatment dose for bovine lipid extract surfactant was 5 mL/kg (135 mg/kg of phospholipids) per dose. Infants who subsequently failed extubation and needed reintubation or remained in 30% oxygen or greater with ongoing radiological evidence of RDS within 72 hours after randomization were eligible for further doses of study surfactant. Repeat doses were provided no more frequently than every six hours, for a maximum of three doses in total. Infants meeting criteria for surfactant beyond 72 hours after randomization or requiring more than three doses of surfactant received bovine lipid extract surfactant or the unit’s standard surfactant as open label. The same allocation was used for all surfactant doses in each patient. Extubation was attempted as soon as possible after administration of the surfactant. Infants were assessed by an RT at 6, 12, 24, 36 and 48 hours to evaluate whether they met extubation criteria.

To ensure blinding, an RT not clinically involved in the care of the baby (for at least the first 48h) was responsible for administering study surfactant at each site. Doses of surfactant were prepared away from the bedside and drawn into a syringe, which was covered by opaque tape, except over the numbers showing the amount in the syringe. Only the RT preparing and administering the study surfactant was therefore aware of the content of the syringe. Administration of surfactant followed a standard process, in order to further ensure blinding and so the process of administration took the same time for all study patients: poractant alfa was administered in a single aliquot, followed by a second air aliquot; bovine lipid extract surfactant was administered in 2 aliquots. Infants in the two groups were treated in an identical manner. The clinicians caring for the patients and the research team remained blinded to the identity of the assigned surfactant throughout the study. This was also applied for any additional dose of surfactant provided to a study patient.

### Outcomes

#### Primary outcome

The primary outcome was alive and extubated at 48 hours post-surfactant administration. Units followed pre-specified guidelines for extubation: rate on ventilator ≤40 per minute, mean airway pressure ≤10 cm H_2_O and fiO_2_ ≤30%.

#### Secondary outcomes

Secondary outcomes included the duration of respiratory support (respiratory support via an endotracheal tube and non-invasive respiratory support), extubation success rates, need for additional surfactant doses, death and pulmonary morbidities up to 36 weeks corrected GA. Pulmonary morbidities included: BPD defined as need for oxygen or respiratory support at 36 weeks corrected GA [12] and duration of oxygen requirement. Secondary outcomes also included adverse events during or within six hours of administration of surfactant: severe airway obstruction (sudden and severe desaturation <80% with heart rate <100 and absence of visualization of chest movement and no response to increases in ventilator pressure or positive pressure inflations for ≥3 minutes and requiring suctioning or changing of endotracheal tube), need for cardiopulmonary resuscitation, pulmonary hemorrhage, and pulmonary air leak.

We also collected data on complications of prematurity: nosocomial infection (defined as positive blood culture or positive cerebrospinal fluid culture), brain injury (intraventricular hemorrhage [IVH] or cystic periventricular leukomalacia present in the worst post-randomization head ultrasound), retinopathy of prematurity (ROP), necrotizing enterocolitis (NEC) (modified Bell staging), metabolic disturbances, patent ductus arteriosus and mortality. All serious adverse events (death, nosocomial infection, NEC stage ≥2 [13], IVH grade ≥3)[14] were reviewed and followed until resolution.

### Statistical analysis

The sample size calculation was based on an anticipated rate of “alive and extubated at 48 hours post-randomization” of 40%, based on our units’ data in 2010. Taking into account changes in extubation patterns, we based our sample size calculation on the percentage of successful extubation within 48 hours in the bovine lipid extract surfactant group lying between 40% and 55%. Fixing the probability of type-I error at 5%, a sample of size of 44 per group provided power in excess of 80% to detect an absolute difference of 30% in the percentage still intubated at 48 hours.

Demographic and clinical characteristics of patients at baseline were summarized by treatment group. Continuous variables were summarized using mean, standard deviation, median, and interquartile range, as appropriate. Discrete variables were summarized using frequency and percent. Analysis of the primary outcome used logistic regression, with treatment group as the main independent variable, and adjusting for the stratification variables, centre, gestational age category and sex. Because the randomization was stratified by centre and gestational age category, the analysis of the primary outcome adjusted for these variables. Additionally, because of an imbalance in gender, this was also adjusted for.

Secondary outcomes were compared similarly. Cox proportional hazards regression was used to examine time-to-event outcomes in order to compare duration of ventilation by treatment group, adjusting for centre, gestational age category and sex. Duration of oxygen support was analyzed similarly. Durations for any patients who died while still on ventilation were treated as censored values. Note that hazard ratios greater than 1 indicate that patients in the poractant alfa group have higher instantaneous risk of experiencing the event of interest. For durations, hazard ratios greater than 1 correspond to *shorter* times. Incidence of adverse events by treatment group were summarized descriptively.

An External Data Safety and Monitoring Board conducted several reviews of patient safety. A formal interim analyses of efficacy and safety took place when 50% of patients were enrolled and their outcome data available. A Haybittle-Peto stopping guideline was set at p<0.001 for each analysis. The study team was not informed of interim results but the DSMB advised the steering committee to continue the study. The analysis was conducted at the end of study only after all efficacy data were complete and finalized.

## Results

**Fig 1:**
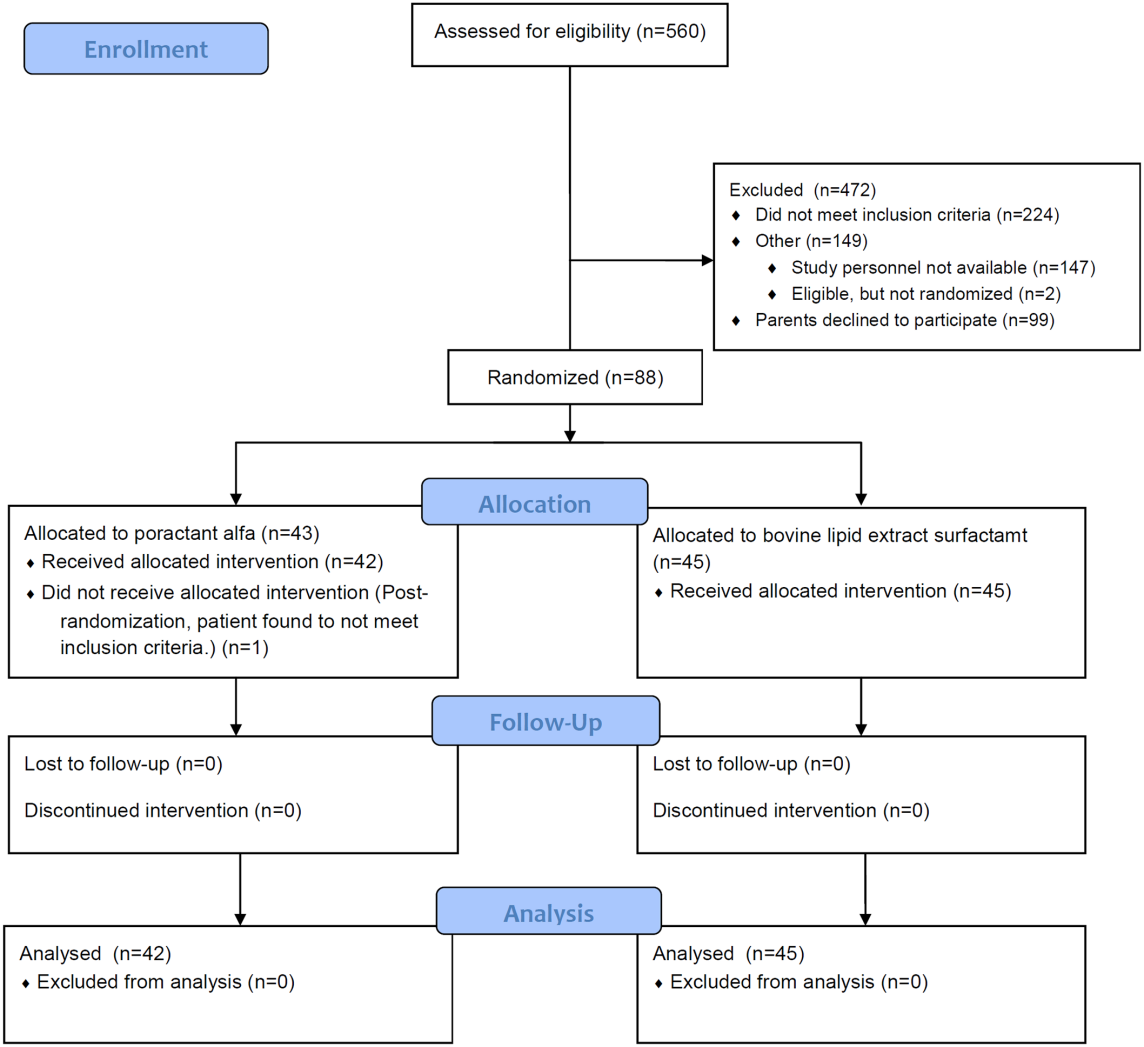
Study flow.

Between March 18, 2013 and December 31, 2015, three sites screened 560 infants, of which 88 were enrolled (Fig 1). Important baseline characteristics were well balanced between groups, with the exception that more male infants were randomized to the bovine lipid extract surfactant group (Table 1). One patient was excluded post-randomization in the poractant alfa group, as he became ineligible shortly after randomization and no longer required surfactant. Data from this patient were excluded from the analysis.

**Table 1:** Baseline characteristics.

Characteristic	Overall (n = 87)	Poractant alfa (n = 42)	Bovine lipid extract surfactant (n = 45)
Male; n (%)	52 (59.8)	19 (45.2)	33 (73.3)
Gestational age (weeks); mean (SD)	26.7 (1.9)	26.5 (1.5)	26.9 (2.1)
24^+0^–26^+6^ GA; n(%)	50 (57.5)	26 (61.9)	24 (53.3)
27^+0^–31^+6^ GA; n (%)	37 (42.5)	16 (38.1)	21 (46.7)
Age at randomization (hours); mean (SD)	5.9 (7.6)	6.3 (7.8)	5.6 (7.5)
Birth weight (g); mean (SD)	906 (272)	880 (202)	931 (324)
Apgar at 1 minute; median (IQR)	4 (3, 6)	4 (2, 6)	4 (3, 6)
Apgar at 5 minutes; median (IQR)	7 (5, 8)	7 (5, 8)	7 (6, 8)
Multiple birth; n (%)	26 (29.9)	12 (28.6)	14 (31.1)
C-section; n (%)	50 (57.5)	25 (59.5)	25 (55.6)
Antenatal glucocorticoids; n (%)			
Complete course	61 (70.1)	28 (66.7)	33 (73.3)
Partial course	25 (28.7)	14 (33.3)	11 (24.4)
None	1 (1.2)	0 (0)	1 (2.2)
Clinical chorioamnionitis; n (%)	11(12.6)	6 (14.3)	5 (11.1)
Premature Rupture of Membrane (> 24h); n (%)	15 (17.2)	11 (26.2)	4 (8.9)
Baseline Fi02; median (IQR)	45 (35, 60) [n = 83]	45 (32, 65)	45 (35, 50) [n = 41]

### Primary outcome

The primary outcome was ascertained in 87 patients (Fig 1). No difference was observed in infants alive and extubated at 48 hours between the study groups: 21/42 (50%) in the intervention group and 26/45 (57.8%) in the control group (adjusted OR 0.76 (95% CI 0.30 to 1.93); p = 0.56) (Table 2). No difference in the primary outcome was observed when examining lower (24–26 weeks) or higher (27–31 weeks) GA strata.

**Table 2:** Primary and secondary outcomes.

Outcome	Overall (n = 87)	Poractant alfa (n = 42)	Bovine lipid extract surfactant (n = 45)	Odds Ratio (95% CI)	Odds Ratio Adjusted for Site, GA, and Gender (95% CI)	P Value
**PRIMARY OUTCOME**						
**Alive and Extubated at 48h n(%)** 24w0d – 26w6d GA 27w0d – 31w6d GA	47 (54) n = 20 n = 27	21 (50) 12/26 (46.2) 9/16 (56.3)	26 (57.8) 8/24 (33.3) 18/21 (85.71)	0.74 (0.32–1.72) 1.67 (0.53–5.25) 0.24 (0.05–1.11)	0.76 (0.30–1.93) 1.74 (0.53–5.73) 0.20 (0.04–1.09)	0.56 0.37 0.06
**SECONDARY OUTCOMES**						
Surfactant doses received; n (%)						
1	70 (80.5)	34 (81.0)	36 (80.0)	1.06 (0.37–3.01)	1.04 (0.35–3.10)	0.94*
2	15 (17.2)	8 (19.1)	7 (15.6)			
3	2 (2.3)	0 (0)	2 (4.4)			
Alive and Extubated at 1 week post-randomization; n (%)	56 (64.4)	29 (69.0)	27 (60.0)	1.47 (0.61–3.55)	1.63 (0.61–4.37)	0.33
Successful first extubation; n (%)**	46/83 (55.4)	22/38 (57.9)	24/45 (53.3)	1.20 (0.50–2.86)	1.16 (0.43–3.13)	0.78
BPD at 36w***; n (%)	48/76 (63.2)	17/33 (51.5)	31/43(72.1)	0.42 (0.16–1.09)	0.35 (0.12–1.04)	0.06
Death; n (%)	12 (13.8)	9 (21.4)	3 (6.7)	3.44 (0.92–12.91)	3.48 (0.94–12.88)	0.06
Death or BPD at 36w; n (%)	59 (67.8)	26 (61.9)	33 (73.3)	0.60 (0.24–1.48)	0.52 (0.19–1.43)	0.21
				**Hazard Ratio** **(95% CI)**	**Hazard Ratio Adjusted for Site, GA, and Gender** **(95% CI)**	**P Value**
Time to first extubation (days); median (IQR)	1.00 (0.47, 3.23)	1.05 (0.51, 2.61)	0.83 (0.47, 5.04)	1.29 (0.83–2.02)	1.21 (0.76–1.94)	0.43
Total duration of respiratory support (days))****						
Mechanical ventilation; median (IQR)	7.87 (1.06, 28.62)	4.12 (1.49, 20.47)	11.93 (0.83, 32.21)	1.38 (0.84–2.27)	1.17 (0.71–1.94)	0.54
Mechanical ventilation (survivors only); median (IQR)	9.76 (1.03, 29.10); n = 75	6.18 (1.49, 22.46); n = 33	11.84 (0.83, 32.21); n = 42	1.12 (0.65–1.92)	0.93 (0.52–1.64)	0.79
nCPAP; median (IQR)	32 (13, 47)	30.5 (11, 44)	40 (21, 48)	1.28 (0.78–2.08)	1.38 (0.80–2.37)	0.25
nCPAP (survivors only); median (IQR)	38 (24, 51); n = 75	36 (27, 51); n = 33	40 (24, 48); n = 42	1.03 (0.60–1.77)	1.07 (0.58–1.97)	0.83
Duration of oxygen use; median (IQR)	45 (27, 76)	41.5 (20, 65)	62 (38, 78)	1.73 (1.06–2.83)	1.69 (1.02–2.80)	0.04
Duration of oxygen use (survivors only) (n = 75); median (IQR)	55 (36, 77); n = 75	51 (34, 65); n = 33	64 (38, 78); n = 42	1.44 (0.84–2.48)	1.38 (0.78–2.45)	0.27

* Comparing the group that received one surfactant dose vs. the group that received 2 or 3 doses

** Event of interest is not being reintubated within 1 week of first extubation. Patients who died during the first intubation period or within a week of the first extubation were excluded from the analysis.

*** One patient was diagnosed with BPD at 36 weeks and died on that day. This patient was included in the analysis for this outcome.

**** Total duration of respiratory support was calculated until death, discharge, or 36 weeks, whichever came first. Note that 16 patients were followed past 36 weeks until discharge, and for these patients, all available data were used to calculate total duration of respiratory support.

### Secondary outcomes

Forty-six infants overall had a successful first extubation (remained extubated for at least one week). No difference was observed between the two groups. The first extubation occurred at a median of 24 hours of life. We observed no difference in the number of infants who required more than one dose of surfactant (Table 2). No statistically significant difference was observed in the duration of intubated respiratory support or the duration of non-invasive respiratory support, in the population overall or in the survivors only. Infants who received poractant alfa spent less time on oxygen. When taking deaths into account, a trend persisted, but it did not reach statistical significance. A trend was observed in the incidence of BPD in survivors, favoring the poractant alfa group: 17/33 (51.5%) vs 31/43 (72.1%), adjusted OR 0.35 (95% CI 0.12–1.04); p = 0.06 (Table 2). No difference was observed in death or BPD at 36 weeks.

Overall, 12 infants died during the study period, 9 in the poractant alfa group (range 1–37 days) and 3 in the bovine lipid extract surfactant group (range 28–78 days). (Tables 2 and 3**)** Five infants in the study had an acute event during or shortly after administration of surfactant; all were randomized to the control group **(**Table 4). All 5 had a severe airway obstruction; two of the five also required cardio-pulmonary resuscitation. These events took place in both GA strata. Ten infants in total developed pulmonary air leak syndromes (either pulmonary interstitial emphysema or pneumothorax) during the study, 4 in the poractant alfa group and 6 in the bovine lipid extract surfactant group. There were no differences in the incidence of IVH, NEC or sepsis between the groups. (Table 5) Fewer infants in the intervention group were diagnosed with ROP (all stages), however there was no difference in the rate of severe ROP between the groups.

**Table 3:** Deaths during study.

Surfactant received	GA at birth (weeks)	Birth weight (g)	Gender	Day of life at death	Cause of death
Poractant alfa					
	26	910	M	1	Pulmonary hypoplasia / pulmonary hypertension
	24	550	F	3	Nosocomial infection
	26	620	M	3	Nosocomial infection
	27	806	M	7	Nosocomial infection
	26	860	F	12	NEC
	24	610	F	19	Nosocomial infection
	25	880	F	22	NEC
	25	850	F	27	NEC
	27	930	M	37	NEC
Bovine lipid extract surfactant					
	26	1040	M	28	NEC
	27	830	M	45	NEC
	24	570	M	78	BPD

**Table 4:** Adverse events.

Outcome	Overall (n = 87)	Poractant alfa (n = 42)	Bovine lipid extract surfactant (n = 45)	Odds Ratio (95% CI)	Odds Ratio Adjusted for Strata (95% CI)	P Value
Adverse events during or after surfactant administration						
Severe airway obstruction; n	5	0	5	0.09 (< 0.01, 1.67)	0.09 (< 0.01, 1.28)	0.07
Need for cardiopulmonary resuscitation; n	2	0	2	0.21 (< 0.01, 4.55)	0.33 (0.03–3.57)	0.36
Pulmonary hemorrhage; n	5	2	3	0.75 (0.14–4.10)	0.87 (0.18–4.31)	0.87
Pneumothorax; n	8	3	5	0.65 (0.16–0.56)	0.59 (0.14–2.51)	0.47
Pulmonary interstitial emphysema; n	2	1	1	1.07 (0.10–11.01)	2.74 (0.28–26.74)	0.39

**Table 5:** Complications of prematurity.

Outcome	Overall (n = 87)	Poractant alfa (n = 42)	Bovine lipid extract surfactant (n = 45)	Odds Ratio (95% CI)	Odds Ratio Adjusted for Strata (95% CI)	P Value
**Complications of Prematurity**						
Nosocomial Infection	34	14	20	0.63 (0.27–1.51)	0.48 (0.18–1.28)	0.14
Intraventricular Hemorrhage (all grades)	35	18	17	1.23 (0.52–2.90)	1.00 (0.39–2.61)	0.99
Stage 3 or higher	9	4	5	0.86 (0.23–3.28)	0.89 (0.23–3.51)	0.87
Periventricular Leukomalacia	1	1	0	3.29 (0.13–86.10)	3.75 (0.33–42.66)	0.29
Necrotizing Enterocolitis (Grade 2 or higher)	11	5	6	0.89 (0.26–3.06)	0.95 (0.27–3.38)	0.94
Retinopathy of Prematurity (All stages)	35 n = 77	11 n = 33	24 n = 44	0.43 (0.17–1.09)	0.13 (0.03–0.58)	< 0.01
Stage 3 or 4	7	3	4	1.03 (0.23–4.60)	0.88 (0.19–4.06)	0.87

We performed a post-hoc analysis, comparing the duration of mechanical ventilation between treatment groups in the two GA strata defined at the onset of the study. This was based on the hypothesis that smaller, more immature infants may demonstrate a different response to surfactant than larger, more mature infants. We found that, in our study, more immature infants (24–26 weeks) who received poractant alfa (n = 26) spent less time on a ventilator than those who received bovine lipid extract surfactant (n = 24): median 9.7 days vs 30.4 days; adjusted OR 2.16 (95%CI1.05–4.43); p = 0.04. When the analysis was repeated to include only infants who survived, infants who were more immature and who received poractant alfa (n = 19) did not spend less time on a ventilator than those who received bovine lipid extract surfactant (n = 22); median 15.6 days vs. 30.4 days; adjusted HR 1.38 (95% CI: 0.61–3.10); p = 0.44.

## Discussion

Our trial is the first to compare bovine lipid extract surfactant, the most commonly used surfactant in Canada, to poractant alfa, which is licensed and used in 60 countries. No difference was observed in our pre-specified primary outcome. Infants who received poractant alfa required less oxygen during their admission. We speculate that the trend observed in BPD and reduction in ROP, favoring the poractant alfa group, likely results from less exposure to oxygen and mechanical ventilation. However, larger randomized controlled trials should examine this further. Although severe ROP was not different between groups, even milder degrees of ROP generate stress and anxiety in parents and thus be considered an important difference. Our results therefore suggest there may be a clinically important difference between the two surfactants. A post-hoc analysis suggests these benefits may be more important in very small and premature infants <27 weeks GA.

Contrary to the meta-analysis of trials comparing poractant alfa to beractant, we did not observe a reduction in the need to re-dose surfactant. This may be because redosing was allowed after 6 hours in both treatment groups in our study, whereas previous studies allowed redosing after 6 hours in the beractant group but only every 12 hours in the poractant alfa group, giving more time for improvement in the infants who received poractant alfa.

We observed 9 deaths in the poractant alfa group and 3 in the bovine lipid extract surfactant group. Although the large majority the deaths were not from respiratory causes, this trend does raise concerns and merits further study. It also limits the assessment of other outcomes competing with death, such as duration of oxygen, duration of respiratory support and BPD.

Poractant alfa has been extensively studied and compared to other animal-derived surfactants. [9,15–17] Findings include a faster onset of action, greater improvement in oxygenation in the first 24-72h, earlier extubation, less redosing and a decreased mortality in infants <32 weeks, when compared to beractant. No differences were found in the incidence of BPD or other complications of prematurity. When compared to synthetic surfactants, poractant alfa was found to have similar efficacy [18] or superior effects (decreased mortality) [19]. No trial reported significant complications during administration.

In the only trial comparing bovine lipid extract surfactant to another animal-derived surfactant, 60 patients (mean GA 27 weeks, 500-1800g with respiratory distress within six hours of life) were randomized to receive either beractant (n = 31) or bovine lipid extract surfactant (n = 29). [6] Thirty-four infants in total were ≤1000g at birth. The study was unblinded and powered to detect a difference in oxygenation indices (OI) between the two groups. Infants in the bovine lipid extract surfactant group had a significantly lower OI throughout the first 8h after the first dose. A reduction in the duration of mechanical ventilation was observed (16.9 vs 19.8 days); no difference was observed in the need to repeat surfactant doses, BPD, CPAP days, oxygen days or hospital stay. This trial was performed more than 10 years ago, when infants were kept on mechanical ventilation longer and when CPAP was not as widely used. No large state-of-the-art study has so far compared bovine lipid extract surfactant to another surfactant following the most modern methods of ventilation and intensive care.

Initial studies reporting the use of bovine lipid extract surfactant did not report complications during administration. [20,21] One unpublished (abstract only) trial comparing bovine lipid extract surfactant to colfosceril palmitate (a synthetic surfactant), reports an incidence of 6% of endotracheal tube obstruction, which was not defined. [22] A recent case series reports an incidence of 20% severe and 28% minor complications during administration of bovine lipid extract surfactant. [7] Five out of six severe airway obstructions occurred in extremely low birth weight infants with more severe lung disease. A standardized protocol for administration of surfactant was created in that institution (McMaster Children’s Hospital) prior to the observational study, to ensure bolus administration in four aliquots, in-line catheter administration and proactive step-wise changes in ventilator settings (pressure and rate) if problems were noticed during administration. Our process of administration of surfactant was standardized in all study centers: we ensured administration in one or two rapid aliquots, which are described as best to improve the distribution in the lungs.[5,23,24] Despite this, we observed five episodes (5/45 = 11%) of severe airway obstruction in infants who received bovine lipid extract surfactant and none in infants who received poractant alfa. They occurred in infants from both GA group strata. None of these five infants died during their admission; all went on to have BPD. We speculate that the higher volume of administration of bovine lipid extract surfactant may explain these events. This observation is concerning and warrants further larger trials.

The strengths of our trial include: 1) treatment allocation was blinded to the clinical and research team; 2) study surfactant administration was standardized and evidence-based and 3) we carefully monitored the clinical course of the infant up to 36 weeks corrected gestational age, to assess moderate term effects. Limitations include the small sample size and the possibility of type 2 errors, which precludes any firm conclusions. Despite pre-specified extubation criteria in each participating center, there may have been infants who met extubation criteria that were not immediately extubated for other clinical reasons. Our definition of BPD was not physiologic therefore did not consider the severity of disease of the infants. Lastly, as we stopped following infants after 36 weeks, we do not have data on duration of NICU, discharge on oxygen, or hospital admission, which would be needed to look at possible costs-benefits.

## Conclusion

No difference was observed in the proportion of infants alive and extubated within 48h between the two study groups. Our trial suggests that poractant alfa may be more beneficial in the treatment of established RDS in premature infants and associated with less risk of complications during administration than bovine lipid extract surfactant. Smaller, more premature infants (<27 weeks) may benefit more than larger infants. A trend towards higher mortality was observed in the poractant group. A larger trial that includes an economic analysis is needed to confirm whether the observed possible benefits translate in shorter hospital admissions, or other long term benefits and determine whether there is a difference in mortality.

## Supporting information

S1 File Study protocol. (DOC)

S2 File CONSORT checklist. (PDF)
