# Supplementary material for: Poractant alfa versus bovine lipid extract surfactant for infants 24+0 to 31+6 weeks gestational age: A randomized controlled trial
Source: PLoS One. 2017 May 4;12(5):e0175922. doi: 10.1371/journal.pone.0175922 (PMC5417427; doi:10.1371/journal.pone.0175922)
Supplement: S1 File — (DOC) [file pone.0175922.s001.doc]

## A randomized, controlled, partially double-blinded, Phase 3, Multi-center trial to determine if Curosurf® reduces the duration of mechanical ventilation in infants 24+0 to 31+6 weeks gestational age

**PRINCIPAL INVESTIGATOR:** Brigitte Lemyre, MD

Children’s Hospital of Eastern Ontario

401 Smyth Road

Ottawa, ON K1H 8L1

(613)737-7600 ext 2415

Email: blemyre@toh.on.ca

#

**Co-Investigators:** Thierry Lacaze-Masmonteil, MD, PhD, FRCPC

Nicole Rouvinez Bouali, MD, FRCPC

Christoph Fusch, MD, FRCPC

Nick Barrowman, PhD, Senior Statistician

Nicole Huneault-Purney, Charge Respiratory Therapist

**Funded By:** Academic Health Science Centers Innovation Fund

**Registered: ClinicalTrials.gov #NCT01709409**

**Current Protocol Version:** Version 2.1, 19Jan2015

# Investigator Agreement

By signing below, I confirm that I have read this protocol and agree to conduct this study in accordance with the procedures described in this protocol, with Good Clinical Practice and with local regulations.

**Name of Principal Investigator (Print)** _________________________________

**Signature of Principal Investigator** _______________________________

**Date** _______________

**Site Address:** The Children’s Hospital of Eastern Ontario

401 Smyth Road

Ottawa, ON K1H 8L1

# List of Abbreviations

ICH International Conference on Harmonization

eCRF Electronic Case Report Form

EDC Electronic Data Capture

GCP Good Clinical Practice

REB Research Ethics Board

REDCap Research Electronic Data Capture

AE Adverse Event

SAE Serious Adverse Event

PI Principal Investigator

ICF Informed Consent Form

LAR Legally Appointed Representative

NICU Neonatal Intensive Care Unit

[A randomized, controlled, partially double-blinded, Phase 3, Multi-center trial to determine if Curosurf® reduces the duration of mechanical ventilation in infants 24+0 to 29+6 weeks gestational age 1](#__RefHeading___Toc320865830)

[Investigator Agreement 2](#__RefHeading___Toc320865831)

[List of Abbreviations 3](#__RefHeading___Toc320865832)

[Study Summary 6](#__RefHeading___Toc320865833)

[1 Background Information and Scientific Rationale 7](#__RefHeading___Toc320865834)

[1.1 Background Information 7](#__RefHeading___Toc320865835)

[1.2 Rationale 7](#__RefHeading___Toc320865836)

[1.3 Potential Risks and Benefits 8](#__RefHeading___Toc320865837)

[2 Study Objectives 8](#__RefHeading___Toc320865838)

[3 Eligibility Criteria 9](#__RefHeading___Toc320865839)

[Inclusion Criteria 9](#__RefHeading___Toc320865840)

[Exclusion Criteria 9](#__RefHeading___Toc320865841)

[4 Study Design 9](#__RefHeading___Toc320865842)

[4.1 Description 9](#__RefHeading___Toc320865843)

[4.2 Study Endpoints 10](#__RefHeading___Toc320865844)

[5 Expected Duration of Subject Participation 10](#__RefHeading___Toc320865845)

[6 Study Medication/Intervention 10](#__RefHeading___Toc320865846)

[6.1 Study Medication Description 11](#__RefHeading___Toc320865847)

[6.2 Control Product Description 12](#__RefHeading___Toc320865848)

[6.3 Formulation, Packaging, and Labeling 12](#__RefHeading___Toc320865849)

[6.4 Accountability Procedures and Storage 13](#__RefHeading___Toc320865850)

[6.5 Subject Compliance with Study Medication/Intervention(s) 13](#__RefHeading___Toc320865851)

[6.6 Concomitant Medications 13](#__RefHeading___Toc320865852)

[7 Study Procedures/Evaluations 14](#__RefHeading___Toc320865853)

[7.1 Screening 14](#__RefHeading___Toc320865854)

[7.2 Baseline time point and Randomization 14](#__RefHeading___Toc320865855)

[7.3 Assessments at 30 minutes, 6 hrs, 12 hrs, 24 hrs, 36 hrs, 48 hrs 14](#__RefHeading___Toc320865856)

[7.4 Week 2, Week 4 and Week 8 15](#__RefHeading___Toc320865857)

[7.5 Discharge home or discharge from the NICU admitting unit 15](#__RefHeading___Toc320865858)

[Timetable of Events 16](#__RefHeading___Toc320865859)

[8 Statistical Plan 17](#__RefHeading___Toc320865860)

[8.1 Sample Size Determination 17](#__RefHeading___Toc320865861)

[8.2 Statistical Methods 17](#__RefHeading___Toc320865862)

[8.3 Interim Analyses 17](#__RefHeading___Toc320865863)

[8.4 Role of the Data Safety Monitoring Board 18](#__RefHeading___Toc320865864)

[9 Safety and Adverse Events 18](#__RefHeading___Toc320865865)

[9.1 Definitions 18](#__RefHeading___Toc320865866)

[9.2 Reporting of Serious Adverse Events 18](#__RefHeading___Toc320865867)

[9.3 Adverse Event Follow-up **Error! Bookmark not defined.**](#__RefHeading___Toc320865868)

[9.4 Treatment Discontinuation 19](#__RefHeading___Toc320865869)

[9.5 Premature Study Discontinuation for an Individual Subject 20](#__RefHeading___Toc320865870)

[9.6 Protocol Violations/Deviations 20](#__RefHeading___Toc320865871)

[10 Data Handling and Record Keeping 20](#__RefHeading___Toc320865872)

[Data Management Responsibilities 20](#__RefHeading___Toc320865873)

[Confidentiality 21](#__RefHeading___Toc320865874)

[Record Retention 21](#__RefHeading___Toc320865875)

[11 Quality Control and Quality Assurance 21](#__RefHeading___Toc320865876)

[Study Monitoring Plan 21](#__RefHeading___Toc320865877)

[Ethical Considerations 21](#__RefHeading___Toc320865878)

[12 Budget & Finance 22](#__RefHeading___Toc320865879)

[13 Publication Plan 22](#__RefHeading___Toc320865880)

[14 References 23](#__RefHeading___Toc320865881)

[15 Appendices 25](#__RefHeading___Toc320865882)

[Appendix A: Time table of Events 25](#__RefHeading___Toc320865883)

[Appendix B: Extubation Criteria 26](#__RefHeading___Toc320865884)

[Appendix C: Extubation Failure Criteria 26](#__RefHeading___Toc320865885)

[Appendix D: Secondary Outcomes 26](#__RefHeading___Toc320865886)

[Appendix E: SOP for study drug administration 27](#__RefHeading___Toc320865887)

[Appendix F: PRODUCT MONOGRAPH-Curosurf® 28](#__RefHeading___Toc320865888)

[Appendix G: PRODUCT MONOGRAPH-BLES® 29](#__RefHeading___Toc320865889)

[Appendix H: Informed Consent 30](#__RefHeading___Toc320865890)

#

# Study Summary

| **Title** | A randomized, controlled, partially double-blinded, Phase 3, Multi-center trial to determine if Curosurf® reduces the duration of mechanical ventilation in infants 24+0 to 31+6 weeks gestational age |
| --- | --- |
| **Short Title** | Curosurf study |
| **Protocol Number** | Curosurf-01 |
| **Phase** | Phase 3 |
| **Methodology** | Randomized, partially double-blinded study using a 1:1 randomization, of ventilated infants to receive either Curosurf® or BLES® by endotracheal method. |
| **Study Duration** | This study is expected to last 30 months, including time for analysis. |
| **Study Center(s)** | Multi-center, multiple sites in Canada |
| **Objectives** | The objective of the study is to determine whether the duration of ventilation in babies who receive Curosurf® is less than those who receive BLES® |
| **Number of Subjects** | The projected enrollment is 88 patients. Enrollment is competitive. |
| **Diagnosis and Main Inclusion Criteria** | Infants born between 24+0 and 31+6 weeks GA, admitted to the study centers NICU’s, with RDS, requiring intubation and surfactant therapy. |
| **Study Product, Dose, Route, Regimen** | Infants will be randomized in a 1:1 manner, to receive either the study drug, Curosurf® or the control drug, BLES®. The treatment dose for Curosurf® is 2.5 ml/kg (200mg/kg) for the first dose and 1.25 ml/kg (100mg/kg) for repeat doses. For BLES® the recommended dose is 5 ml/kg. Either drug is given by syringe into the endotracheal tube by a Respiratory Therapist. |
| **Duration of administration** | The study drug or control drug will be given once all inclusion and no exclusion criteria have been met. In the days to follow according to the status of the infant, repeated doses may be indicated. |
| **Reference therapy** | The control group will receive BLES®. This licensed medication is routinely used in both study centers for the treatment of ventilated infants with a diagnosis of RDS. |
| **Statistical Methodology** | Analysis of the primary outcome will use logistic regression, with the treatment group as the main independent variable, and adjusting for the stratification variables, centre and gestational age category. Secondary analyses will include comparison of mortality rates by treatment group using a similar logistic regression model. |

# Background Information and Scientific Rationale

## Background Information

Evidence for the efficacy of either prophylactic (within the first 30 minutes after birth regardless of respiratory status) or selective (when signs of respiratory distress syndrome or RDS are present) administration of surfactant comes from meta-analyses, which demonstrate a consistent 30-50% reduction in the odds of neonatal death and air leaks (1). As the increase in survival is mainly observed among extremely low birth weight (ELBW) infants, the incidence of bronchopulmonary dysplasia (BPD) - the chronic lung disease that follows ventilator and O_2_ therapy for RDS - has not been significantly reduced. BPD, defined by the need for supplemental O_2_ at 36 weeks post menstrual age (PMA), increases the risk of mortality and pulmonary and neurodevelopmental morbidity among ELBW infants (2). Recent large RCTs have demonstrated that early nasal continuous positive airway pressure (nCPAP) with selective surfactant therapy was as effective at reducing BPD or death as intubation and prophylactic surfactant (3-5).

Currently, the two surfactants available in Canada are BLES® (Bovine Lipid Extract Surfactant) purified from bovine lung lavage and beractant (Survanta) extracted from minced bovine lungs. Most neonatal intensive care units (NICUs) use BLES®, for its observed faster onset of action. The recommended dose is 5 ml/kg, the highest for an animal-derived surfactant. Recent observations of severe airway obstruction (SAO) with desaturation and/or bradycardia with BLES® were reported in a cohort of premature infants, with an incidence of SAO of 31% in ELBW infants (6).

In Europe and the USA, the most widely used surfactant is poractant alfa (Curosurf®), a surfactant extracted from porcine minced lungs. Compared to BLES® and Survanta, Curosurf® contains a higher concentration of phospholipids (27, 25, and 76 mg/ml respectively). This accounts for the dramatic improvement usually observed immediately after its administration, which translates into a higher rate of extubation success (7-9). The treatment dose is 2.5 ml/kg (200mg/kg) for the first dose and 1.25 ml/kg (100mg/kg) for repeat doses if indicated. A recent meta-analysis confirms that Curosurf®, compared to Survanta, leads to higher survival, shorter duration of mechanical ventilation, less re-dosing (needing > 1 dose of surfactant) and shorter admissions to NICU (10).

Current practice in Canada is to selectively intubate patients who demonstrate signs of RDS and provide them with BLES®, with the aim of extubating to nCPAP as soon as possible, in order to avoid or reduce lung injury. Despite our intent, the mean duration of intubation after surfactant for <30 weeks gestational age (GA) survivors in Ottawa was 6.8 days in 2010. In the same year, 60% of infants <30 weeks GA who received BLES® in Ottawa remained ventilated at 48 hours. Recent trials using Curosurf® report median durations of intubation of 2-5.5 days in infants <29 weeks GA as well as rates of BPD (21% in the CURPAP study) lower than the current incidence in Canada (44.8%) (7,8,11).

## Rationale

Recent studies in Europe and Australasia investigated the possibility of instilling surfactant directly in the trachea with a thin, soft tube under direct visualization, to avoid intubation and mechanical ventilation completely (11,12). These have shown feasibility and German investigators have reported very low rates of death or BPD (15%) in infants 26-28 weeks GA with that strategy (11). Discussions about adopting this approach are ongoing in Ontario and Canada but neonatologists are skeptical as to whether this would be possible using BLES®.

In Canada, our initial respiratory management changed in the last decade, however the surfactant used has remained unchanged since the late 1990’s. In the only published (abstract form only) RCT comparing BLES® to Exosurf, a synthetic surfactant no longer used in North America, no difference was found in the primary outcome (survival without BPD at 36 weeks PMA). As many secondary outcomes favored BLES®, the investigators concluded that BLES® was a superior surfactant (13). BLES® was compared to Survanta in only one small RCT. Patients who received BLES® achieved a faster clinical response in terms of oxygenation index. Mortality, ventilator days and chronic lung disease were not different between groups (14). There is no published study comparing BLES® to Curosurf®. This RCT will be the first clinical trial comparing Curosurf® to BLES® and the first use of Curosurf® in Canada. Introducing Curosurf® in Canada for the smallest of our infants might lead to changes in the approach to initial respiratory management translating to a better respiratory outcome.

## Potential Risks and Benefits

**Potential Benefits**

The potential benefit of this trial is that Curosurf® may reduce the time that the infant is exposed to mechanical ventilation, thereby potentially reducing the complications associated with intubation and ventilation, such as pneumonia and barovolutrauma. As well, Curosurf® is a more concentrated medication, therefore smaller volumes are administered. Infants may therefore have fewer complications (such as desaturations, apnea and bradycardia) due to airway obstruction in the period immediately following the administration.

**Potential Risks**

Administration of surfactant produces a transient decrease in ventilation (as surfactant partially occludes the airway) and oxygenation and may be associated with a pulmonary air leak (pneumothorax or pneumomediastinum) as compliance acutely improves. Pulmonary hemorrhage and intraventricular hemorrhages have been associated with surfactant administration as well. These risks are independent of the type of surfactant used.

For a detailed list of AEs associated with the study drugs please see the Drug Monographs (Appendices F and G). Please also note that many of the AEs listed for these drugs are in fact complications of prematurity but due to reporting regulations these AEs are listed in the drug AE section of the monographs.

# Study Objectives

The objective of the study is to compare the efficacy of the 2 surfactants Curosurf® and BLES®, at facilitating extubation and reducing pulmonary morbidities and also to compare the adverse events during surfactant administration in very premature infants. We hypothesize that, compared to BLES®, Curosurf® will increase the proportion of infants alive and extubated within 48 hours after surfactant therapy.

**Primary objective**

The primary objective of the study is to compare between the two groups, the number of subjects alive and extubated at 48 hours post surfactant administration.

**Secondary objectives**

To compare the duration of respiratory support, extubation failure rates, need for additional surfactant doses, adverse events (during and following administration), survival and pulmonary morbidities during hospital admission between the two groups.

# Eligibility Criteria

## Inclusion Criteria

- 1. Infants born between 24+0 and 31+6 weeks gestational age, admitted to the study centers
  2. Infants with RDS requiring intubation and surfactant therapy within 48 hours after birth

## Exclusion Criteria

- 1. Any infant more than 48 hours of age
  2. Any infant with a pulmonary hemorrhage
  3. Any infant with life-threatening congenital anomaly or one that is considered non-viable
  4. Any infant on rescue high frequency ventilation
  5. Any infant known to require early intubation and ventilation for surgical treatment of a congenital anomaly
  6. Any infant with anomalies of the upper or lower airway or mandible precluding use of nCPAP
  7. Any infant born after prolonged premature rupture of membranes (<22 weeks GA or >28 days prior to delivery)
  8. A parent/LAR who is incapable of, or unwilling, to give consent
  9. Participation in another clinical trial of any placebo, drug, biological, or device conducted under the provisions of a protocol
  10. Any other reason as deemed significant by the Investigator

# Study Design

## Description

This is a Phase 3 randomized, controlled, partially double-blinded trial. Infants with a diagnosis of RDS will be randomly assigned in a 1:1 manner to receive either the study drug, Curosurf® or BLES® (control group), by endotracheal method. A total of 88 infants will be in Canada. The study will take place at multiple sites. Enrollment is competitive.

Each infant enrolled into the study will receive a unique subject number. Subject codes will not be reused and each infant can be randomized only one time for the entire trial period.

The expected duration for each subject/infant is dependant on the gestational age of the infants at birth, as all babies will be followed until 36 weeks gestational age (GA) (or until discharge, which ever comes first), thus the longest duration for one infant should be 12 weeks.

Following the baseline and randomization time point there are a maximum of 6 assessment time points, starting at 30 minutes post study medication administration. The infants will be assessed by the Respiratory Therapist (RT) and the physician to determine whether or not they meet extubation criteria and can be extubated. Follow-up to collect information about the duration of ventilation will then occur at 2 weeks, 4 weeks and 8 weeks. The final time point for data collection is at 36 weeks gestational age or at discharge, whichever occurs first.

The entire trial should be completed within 30 months.

This will be a Regulated Trial and a Clinical Trial Application to Health Canada will be completed prior to beginning the trial. Local Research Ethics Board approval will be obtained at all sites, again prior to study start up.

## Study Endpoints

**Primary Endpoint**

The primary efficacy endpoint is that the patient is alive and extubated at 48 hours post surfactant administration. We hypothesize that Curosurf® will increase the proportion of infants alive and extubated within 48 hours after surfactant therapy.

**Secondary Endpoints**

The following endpoints will also be measured in this study:

- duration of respiratory support (respiratory support via an endotracheal tube and non-invasive respiratory support)
- duration of NICU stay
- extubation failure rates
- need for additional surfactant doses
- adverse events (during and following administration of surfactant)
- death before discharge
- incidence of pulmonary morbidities up to 36 weeks GA

# Expected Duration of Subject Participation

The duration for each infant in the study will vary according to the gestational age. Follow up is done until the infant is 36 weeks GA or at discharge, whichever is first. All assessments will be done according to the Timetable of Events. These time points are simply for data collection and no extra tests or procedures are done for the purposes of this study, from the perspective of the infant or the parent/LAR.

# Study Medication/Intervention

Infants will be randomized in a 1:1 manner, to receive either the study drug, Curosurf® or the control drug, BLES®. The treatment dose for Curosurf® is 2.5 ml/kg (200mg/kg) for the first dose and 1.25 ml/kg (100mg/kg) for repeat doses. For BLES® the recommended dose is 5 ml/kg. Either drug is given by syringe into the endotracheal tube by a Respiratory Therapist. The SOP for administering both study drugs is found in Appendix E of this protocol.

After consent is obtained, and all inclusion/exclusion criteria are fulfilled an infant can be randomized into the trial. A blinded study RT will administer the drug according to the SOP found in Appendix E (also in the Resource Manual). The RT will document the procedure using the source documents provided by the sponsor and will also complete all Drug Accountability logs, as appropriate. Detailed instructions for completion of drug accountability logs are found in the Resource Manual.

The blinding procedure for administration of both study drugs is as follows:

- As the volume and viscosity of Curosurf® and BLES® are different, blinding of the RT administering the surfactant is not feasible. However, at any given time, two RTs work in the NICU. Doses of surfactant will be prepared away from the bedside by a RT not otherwise involved in the care of the infant. The surfactant will be drawn into a syringe and placed in a covered box. The RT will administer the surfactant with the syringe masked by a large label covering the amount of the contents. The RT will record in the patient’s chart that surfactant was provided but will not include the name or the dose of the product. This RT will not care directly for the infant for at least 48 hours following administration of the surfactant. The clinical and research teams will be blinded to the identity of the assigned surfactant throughout the study, unless an emergency occurs requiring unblinding

## 6.1 Study Medication Description

Study medication for this trial will be maintained in bulk supply format, the vials will not be individually numbered. For both Curosurf® and BLES®, the correct dose, which is weight based, will be calculated by the study Investigator and the RT will be responsible to double check the calculation. Either study drug is given by endotracheal method within 48 hours of life. Repeated doses may be given, up to a maximum of 3. If further doses are required then open label BLES® should be administered. The doses should not be given more frequently than every 6 hours.

**Adverse Reactions**

Both study drugs have potential adverse reactions and the study staff should carefully monitor the infant for any or all of the following:

**Adverse Reactions of both Curosurf**® **and BLES**®

- Bradycardia and hypotension -during administration

**Adverse Reactions of Curosurf**®

- patent ductus arteriosus
- intraventricular hemorrhage of all grades,
- pneumonia
- sepsis
- pulmonary interstitial emphysema,
- pulmonary hemorrhage
- pneumothorax
- recurrent apnea
- retinopathy of prematurity,
- bronchopulmonary dysplasia

**Adverse Reactions of BLES**®

*Very common adverse* events occurring in10% of infants who received BLES® (bovine lipid extract surfactant), in descending order of frequency, were:

- patent ductus arteriosus
- decreased post-dose pulmonary function values,
- intraventricular hemorrhage of all grades,
- sepsis,
- retinopathy of prematurity,
- bradycardia
- severe intraventricular hemorrhage

*Common adverse events* occurring in1% and < 10% of infants who received BLES®, in descending order of frequency, were;

- pulmonary interstitial emphysema,
- periventricular leukomalacia,
- pneumothorax,
- pulmonary hemorrhage,
- endotracheal tube complications,
- necrotizing enterocolitis,
- respiratory acidosis,
- convulsions,
- hypotension,
- apnea,
- hydrocephalus
- pneumonia

.

## 6.2 Control Product Description

BLES® is available in 3 and 5 ml vials for this trial. The vials are stored in a -20 degrees Celsius freezer for long term storage. A two month supply will be thawed and kept in the refrigerator, so that both study drugs will come from the same storage conditions in order to maintain blinding procedures. The drug, once thawed is opaque and milky in appearance.

## 6.3 Formulation, Packaging, and Labeling

Curosurf® is manufactured and provided by Chiesi Farmaceutici S.p.A. A detailed Product Monograph is found in Appendix F. The vials will be labeled for investigational study use. The commercial label will not be present. The vials will be shipped and stored between +2 and +8 degrees Celsius. There are two vial sizes for the study 1.5 mls and 3.0 mls.

The label applied on the vial will contain the following information:

| Curosurf-01  Poractant alfa 80 mg/mL - X mL  Expiration: DD-MMM-YYYY  Store in refrigerator between +2°C and +8°C, protected from light  Lot # -------  Protocol Code: ----------  Sponsor: The Ottawa Hospital, 501 Smyth Road, Ottawa ON K1H 8L6  Investigational drug to be used only by a qualified Investigator | Curosurf-01  Poractant alfa 80 mg/mL - X mL  Expiration: DD-MM-YYYY  Conserver au réfrigérateur entre +2°C et +8°C, à l’abri de la lumière  Lot # -------  Code du Protocole: ------------  Commenditaire: The Ottawa Hospital, 501 Smyth Road, Ottawa ON K1H 8L6  Médicament expérimental à être utilisé seulement par un Investigateur qualifié |
| --- | --- |

BLES® is manufactured by BLES Biochemicals Inc. A detailed Product Monograph is found in Appendix G. A dedicated stock of 3 and 5 ml vials will be secured by the sponsor. The vials will be relabeled using the template as below.

| Curosurf-01  BLES- X mL  Expiration: DD-MMM-YYYY  Store at -20°C for long term and then may be thawed and stored at +2°C to +8°C  Lot # -------  Sponsor: The Ottawa Hospital,  501 Smyth Road, Ottawa ON K1H 8L6  Investigational drug to be used only by a qualified Investigator | Curosurf-01  BLES- X mL  Expiration: DD-MM-YYYY  Conserver au congélateur à long-terme à -20°C et peut ensuite être dégelé et conservé de nouveau entre +2°C et +8°C  Lot # -------  Commenditaire: The Ottawa Hospital,  501 Smyth Road, Ottawa ON K1H 8L6  Médicament expérimental à être utilisé seulement par un Investigateur qualifié |
| --- | --- |

## 6.4 Accountability Procedures and Storage

**Investigational Group-Curosurf**®- Vials will be stored between +2 and +8 degrees Celsius. The study medication will be received by the site pharmacy and a drug receipt form will be completed and sent back to the shipper. A two month supply of drug will be transferred to the NICU where it will be stored in a locked container.

**Control Group-BLES**®- The vials will be stored in a -20 degrees Celsius freezer in the study center Pharmacy. A two month supply will be thawed and kept in the refrigerator in the NICU, in a dedicated locked container, to avoid mixing with regular NICU stock. These will be clearly labeled with an “expires on ____” date, to avoid administering drug that has been at refrigerator temperatures over the allotted time.

For both study drugs temperatures will be monitored daily, both in the main pharmacy and in the NICU, capturing the minimum and maximum temperatures for the previous 24 hours. Detailed instructions for cold chain management are found in the Study Resource Manual.

Drug accountability forms will be completed in the pharmacy for the main stock and subject accountability forms will be completed as each vial is administered in the NICU. Please see the Resource Manual for further instructions for completing the Drug Accountability forms.

## 6.5 Subject Compliance with Study Medication/Intervention(s)

Not applicable for this study.

## 6.6 Concomitant Medications

Narcotics/opiods will be the only concomitant medications collected for this trial. Any medications in this category given within 6 hours of study medication initiation up to 6 hours post extubation will be recorded, except in case of an SAE (where all con meds for the SAE will be captured).

# Study Procedures/Evaluations

There are minimal study procedures for this trial. The length of participation varies for each infant, depending on the gestational age at enrollment, therefore the number of data collection time points will also vary. The baseline, randomization and assessments while on the ventilator are standard for all subjects but the number of follow up time points will vary. A Timetable of Events follows on a subsequent page for a summary of all the study time points and activities.

## 7.1 Screening

There will be no screening time point for this study.

## 7.2 Baseline time point and Randomization

The baseline time point will occur after the consent has been obtained and the infant’s status fulfills the inclusion criteria, and no exclusion criteria. The following procedures will occur:

- Physical exam (includes vital signs) (the infant will not be reweighed for study purposes, the weight will be taken from the medical history)
- Medical History
- Concomitant medication history

The infant can then be randomized into the study. Each infant will be allocated to receive either Curosurf® (study drug) or BLES® (control) in a 1:1 ratio using a computerized random-number generator. Twins or higher multiples will be randomized as individual patients. Randomization will be stratified by center and by GA category (24-26 weeks and 27-31 weeks), with random block lengths. Allocation will be concealed in sealed, opaque, sequentially-numbered envelopes, which will be opened by a Respiratory Therapist (RT) not involved in the care of the infant. This will occur only after intubation. The drug will be prepared by the RT. Blinding procedures will occur as per Section 6 of this protocol.

A maximum of 3 doses can be given over a 72 hour period post randomization. Infants not extubated and requiring ≥30% oxygen with radiological evidence of RDS can receive further dose(s) of surfactant. Similarly, infants who fail extubation and need to be reintubated (criteria in Appendix C) could receive further dose(s) of surfactant if they require ≥30% oxygen with ongoing radiological evidence of RDS. The same blinding method will be used for each dose of surfactant given and each dose of surfactant will follow the randomization sequence (i.e. once randomized, an infant will receive the type of surfactant that was given at randomization).

## 7.3 Assessments at 30 minutes, 6 hrs, 12 hrs, 24 hrs, 36 hrs, 48 hrs

Every infant will have an assessment starting at 30 minutes, next at 6 hours, 12, hours, 24 hours, 36 hours, and 48 hours, while still intubated. At these designated times the infant will be assessed to see if they can be extubated using the criteria listed below (also Appendix B).

Once the infant has been extubated then the assessments for extubation will no longer be performed. If at any point the infant fails extubation and needs to be reintubated, this information will be captured and recorded at the next time point for data collection.

**Extubation criteria**

Infants enrolled in the study will be extubated when they meet all of the following criteria:

1. rate on ventilator ≤40 per minute and
2. mean airway pressure ≤ 10 cm H_2_0 and
3. fi0_2_ ≤ 30%

At these time points, information about SAEs, ventilator parameters, and con meds will also be collected.

**7.3.1 Reintubation and further drug administration**

If an infant has failed extubation and requires reintubation, this information will be captured at the subsequent study timepoint. Further doses of surfactant can be given, if the maximum (3 doses) has not been already administered, up to a maximum of **72 hours** post randomization.

If the study Investigator deems that a subject needs further doses of surfactant beyond 72 hours post-randomization then this subject will be removed from the study and followed as per section 9.4 of this protocol.

## Week 2, Week 4 and Week 8

At weeks 2, 4 and 8 (if the infant has not been discharged) any information pertaining to SAEs (if within the 30 day follow-up for SAEs) and any extubation assessment data should be captured. If any infant that has been transferred to another institution the site must make every effort to follow the infant. This should be done in accordance with the local Ethics Boards guidelines and approval should be requested as necessary.

## Discharge home or discharge from the NICU admitting unit

This time point can occur at 36 weeks gestation or before. All SAEs con meds, extubation failure and secondary outcome assessments data will be captured (if not previously done). Any SAE information will be gathered and completed, unless the SAE has not resolved.

| Timetable of Events | | | | | | | | | | | |
| --- | --- | --- | --- | --- | --- | --- | --- | --- | --- | --- | --- |
| **Evaluation** | **Baseline** | **30 m.**  **(± 15 min)** | **6 hrs**  **(± 30 min)** | **12 hrs**  **(± 2 hrs)** | **24 hrs**  **(± 2 hrs)** | **36 hrs**  **(± 2 hrs)** | **48 hrs**  **(± 2 hrs)** | **Week 2**  **(±1 week)** | **Week 4**  **(±1 week)** | **Week 8^*^**  **(±1 week)** | **Discharge from hospital**  **(36 wksGA)** |
| Consent ^1^ | X |  |  |  |  |  |  |  |  |  |  |
| Medical/Medication History | X |  |  |  |  |  |  |  |  |  |  |
| Physical Exam ^2^ | X |  |  |  |  |  |  |  |  |  |  |
| Demographics | X |  |  |  |  |  |  |  |  |  |  |
| Randomization | X |  |  |  |  |  |  |  |  |  |  |
| Ventilator parameters^3^ | X | X | X | X | X | X | X |  |  |  |  |
| Con Meds | X |  |  |  |  |  | X | X | X | X | X |
| SAE assessment |  | X | X | X | X | X | X | X | X | X | X |
| Extubation failure assessment |  |  |  |  |  |  |  | X | X | X | X |
| Secondary outcome assessment |  |  |  |  |  |  |  |  |  |  | X |

^1^ Consent may be obtained from the mother antenatally or after the birth of the infant.

^2^ Includes weight, Temperature (Axillary), heart rate (apical), respirations.

^3^ Includes Rate, PL-LP, PEEP, Pressure support, FiO2, Mean, Tidal volume, IT, and Flow

**^*^** Data collection at this time point is not done if the infant has already been discharged.

.

# Statistical Plan

## 8.1 Sample Size Determination

Review of our data on duration of ventilation indicates that in 2010, 40% of patients meeting inclusion criteria were alive and extubated 48 hours after BLES®. We anticipate that the mean length of ventilation in the BLES® group will be similar or shorter in 2012. We therefore based our sample size calculation on the percentage of successful extubation within 48 hours in the BLES® group lying between 40% and 55%. Fixing the probability of type-I error at 5%, a sample of size of 44 per group will provide power in excess of 80% to detect an absolute difference of 30% in the percentage still intubated at 48 hours. (See Table)

| Percentage alive and extubated at 48 hrs | |  |  |
| --- | --- | --- | --- |
| BLES® group | **Detectable in Curosurf® group** | Risk difference | Relative risk reduction |
| 40% | **69.3%** | 29.3% | 0.488 |
| 45% | **73.9%** | 28.9% | 0.525 |
| 50% | **78.3%** | 28.3% | 0.566 |
| 55% | **82.3%** | 27.3% | 0.607 |

A recent RCT in a similar population found that 52% of infants who received Curosurf® were extubated by 48h compared to 22% of infants who received Survanta, an absolute reduction of 30% (15).

## 8.2 Statistical Methods

Demographic and clinical characteristics of patients at baseline will be summarized by treatment group. Continuous variables will be summarized using mean, standard deviation, median, and interquartile range, as appropriate. Discrete variables will be summarized using frequency and percent. Analysis of the primary outcome will use logistic regression, with treatment group as the main independent variable, and adjusting for the stratification variables, centre and gestational age category. The intention to treat principle will be followed. Secondary analyses will include comparison of mortality rates by treatment group using a similar logistic regression model. Use of oxygen at 36 weeks will be analyzed similarly. Cox regression will be used to compare duration of ventilation by treatment group, adjusting for centre and gestational age category. Duration of oxygen support will be analyzed similarly. Durations for any patients who die while still on ventilation will be treated as censored values. Incidence of adverse events by treatment group will be summarized descriptively.

## Interim Analyses

Once 50% of patients have been recruited or 1 year has passed, whichever is sooner, the study statistician will perform a blinded descriptive analysis of participants’ baseline characteristics and safety data, which will be reviewed by the Data Safety Monitoring Board (DSMB).

## 8.4 Role of the Data Safety Monitoring Board

This study will be monitored by an independent Data Safety Monitoring Board (DSMB), consisting of an independent physician, and two other members not involved in this study. The DSMB will be immediately informed of any serious adverse events (SAEs), both related and unrelated,. If at any point the DSMB considers continuance of the study unacceptable the steering committee will be immediately notified.

Interim reports, prepared by the data management team for the study, for review by the DSMB will include data on recruitment, compliance, adverse effects, baseline comparability and treatment comparisons. An agreed upon review package which contains the appropriate data summary by treatment will be provided by the study statistician for the purposes of these reviews. DSMB members will review data only by masked study group (such as A vs. B rather than experimental vs. control). The DSMB may request unblinded safety data tables, in case of adverse events associated with study drug or procedures.

Serious adverse events, which are of concern to members of the Steering Committee (potentially study drug related) will also be reviewed by the DSMB.

Members of the DSMB will also be responsible for developing terms of reference including clinical stopping rules. If at any point the DSMB considers continuance of the study unacceptable, the steering committee will be immediately notified.

# Safety and Adverse Events

The study Investigators will comply with ICH Guidelines, as per GCP section 4.11, Health Canada Regulations, and their local REB requirements to submit any new adverse events that are both serious and unexpected. Any new information regarding the safety of research subjects should also be submitted.

## 9.1 Definitions

Serious Adverse Event (SAE)

Adverse events are classified as serious or non-serious. A serious adverse event is any AE that is:

- fatal
- life-threatening
- requires or prolongs hospital stay
- results in persistent or significant disability or incapacity
- a congenital anomaly or birth defect
- an important medical event

Important medical events are those that may not be immediately life threatening, but are clearly of major clinical significance. They may jeopardize the subject, and may require intervention to prevent one of the other serious outcomes noted above.

## 9.2 Reporting of Serious Adverse Events

For this study only Serious Adverse Events will be reported.

A related serious adverse event must be reported to the sponsor, the local REB and the applicable Regulatory body (Health Canada) as soon as possible but no later than 7 calendar days after first knowledge of the event. Further information and significant new information on ongoing serious adverse events will be provided to the sponsor, the REB and Health Canada. Copies of all information about the SAE will be kept in the Regulatory binder.

All SAEs will be followed until resolution or until the Principal investigator is of the opinion that the SAE has stabilized and no more follow-up is required. SAEs will be reported using the REDCap EDC system. The SAE must be entered into the REDCap SAE reporting page within 24 hours of learning of the event. Once the information is entered an email will be automatically sent to the Principal Investigator informing her of the event.

**Morbidities or Complications of prematurity**

- Air leaks (pneumothorax, pneumomediastinum, pneumopericardium, pneumoperitoneum)
- Necrotizing enterocolitis (NEC) of stage 2 or greater as per modified Bell’s criteria (16)
- Patent ductus arteriosus (PDA) documented by echocardiography, medically treated / surgically ligated / haemodinamically significant
- Pulmonary interstitial emphysema
- Pulmonary hemorrhage
- Intraventricular hemorrhage according to Papile’s classification (17)
- Periventricular leukomalacia (PVL) identified by cranial ultrasound(18)
- Retinopathy of prematurity (ROP) (19)
- Bacterial sepsis and/or meningitis
- Bronchopulmonary dysplasia (BPD) (20)

Presence of the following abnormal laboratory values:

- hypoglycemia
- hyperglycemia
- hypocalcemia
- hypercalcemia
- hyponatremia
- hypernatremia

The complications of prematurity, listed above, are clinically expected outcomes in preterm neonates aged between 24 and 32 weeks, therefore not subject to expedited reporting.

However, a worsening of these clinical outcomes that occurs after study drug administration will be recorded as ADRs if related to study drug and subject to expedited reporting if serious.

The complications of prematurity will be recorded in the electronic case report form (eCRF) as clinical documentation of each neonate. Specifically the details are as follows:

- Presence / Absence
- Start date and start time
- Ongoing / Solved
- If solved, End date and end time
- In case of sepsis, also number of occurrences

## 9.4 Treatment Discontinuation

The criteria for permanent discontinuation of further study product/interventions for an individual subject are as follows:

- Completion of treatment/intervention as defined by the protocol
- Clinical reasons believed to be life-threatening by the physician
- SAE occurring during and shortly after administration of the study drug

The infant will continue to be followed with the parent’s/LAR’s permission if the study treatment/intervention is discontinued. There will be no changes to the follow-up time point schedule, except no study treatment/intervention will be administered.

## 9.5 Premature Study Discontinuation for an Individual Subject

The criteria for permanent discontinuation from the study for an individual subject are as follows:

- Request of the infant’s parent/LAR to withdraw from the trial
- Any clinical adverse event, laboratory abnormality, intercurrent illness, other medical condition or situation occurs such that continued participation in the study would not be in the best interest of the infant.

In the event that the infant is withdrawn from the study due to an SAE, this will be recorded in the eCRF and in the source documentation. The infant will be followed and treated by the Investigator until the abnormal parameter or symptom has resolved or stabilized. It will be up to the clinician to determine that the SAE is either resolved or that it has reached a stable state, after which no further follow-up is necessary. There will be source documentation to support this determination.

## 9.6 Protocol Violations/Deviations

Protocol violations/deviations will be reported by all study sites. Each site will keep a log of protocol violations/deviations that will be compiled at the end of the study. Each site will be responsible to report violations and deviations to their respective REBs, as per local requirements.

If any subject receives the incorrect study drug, this information will be reported to the sponsor.

If an infant requires more than one dose of study medication and it is discovered that the incorrect drug has been dispensed then this will be reported. The infant will be removed from the study but will be followed until the last timepoint of the study for safety reasons.

# Data Handling and Record Keeping

## Data Management Responsibilities

Data collection will be the responsibility of the research staff at each site under the supervision of the Principal Investigator and Sub-Investigators. During the study the investigator will maintain complete and accurate documentation for the study.

Data collection for the study will be done using an eCRF (REDCap). Both study centers will enter study information into this system. All information will be de-identified, using a unique subject number for each infant enrolled into the study.

All source documents and lab reports must be reviewed by the clinical team and data entry staff, who will ensure that they are accurate and complete. Adverse events will be graded, assessed by severity and causality and reviewed by the site investigator or designee.

## Confidentiality

All subject related information including Case Report Forms, evaluation forms, reports, etc. will be kept strictly confidential. All records will be kept in a secure, locked location and only research staff will have access to the records. Subjects will be identified only by means of a coded number specific to each subject. All computerized databases will identify subjects by numeric codes only, and will be password protected or encrypted.

Upon request, subject records will be made available to the study sponsor, monitoring groups’ representative of the study sponsor, and applicable regulatory agencies such as Health Canada.

## Record Retention

All research records for this trial (as it falls under Health Canada Division 5 regulations) will be retained for a minimum of 25 years after study closure.

# Quality Control and Quality Assurance

The trial will be conducted in accordance with the latest version of the Declaration of Helsinki, GCP, ICH regulatory guidelines, and Division 5 of Health Canada Food and Drug regulations and requirements regarding ethical committee review, informed consent, and other statutes and regulations regarding the protection of the rights and welfare participants participating in the study.

The protocol, including the informed consent document and all recruiting materials, will be submitted to the Research Ethics Boards for review and approval. No changes will be made to the protocol without REB approval, except where necessary to eliminate apparent immediate hazards to participants. The parent/LAR will be able to withdraw their consent to participate at any time without prejudice. Additionally, the investigators may withdraw an infant if, in the investigator’s clinical judgment, it is in the best interest of the infant.

Data will be collected using REDCap EDC. This system will be used to generate reports. There is also an audit trail function which is compliant with current Canadian regulations.

Source documents (version controlled) will be provided by the sponsor. These documents will be monitored by a representative of the sponsor.

## Study Monitoring Plan

On-site monitoring will be conducted by qualified research personnel. The Monitoring time points will occur approximately every 6-12 months or as needed, based on enrollment and subject study time points. The Essential Documents in the Investigator Regulatory Files will be monitored using the Monitoring Timepoint Report template. The monitor will identify any items missing from the Regulatory Binder. Site personnel are responsible for maintenance of the Regulatory Binder. The consent document will be reviewed for content to ensure it contains the required (and additional, as applicable) regulatory elements. The consent document will be compared to the protocol and site specific REB procedures for informed consent documentation to ensure agreement between the two documents. Consent form monitoring will be documented in the monitoring time point report.

## Ethical Considerations

This study will be conducted according to Canadian and international standards of Good Clinical Practice for all studies. Applicable government regulations and local study center research policies and procedures will also be followed.

This protocol and any amendments will be submitted to local REB for formal approval to conduct the study. The decision of the REB concerning the conduct of the study will be made in writing to the investigator.

All parents of infants for this study will be provided a consent form, describing the study and providing sufficient information for them to make an informed decision about their infant’s participation in the study. The consent form will be submitted with the protocol for review and approval by the REB. The formal consent of a subject, using the REB-approved consent form, will be obtained before the infant is submitted to any study procedure. This consent form must be signed by the infant’s parent or legally acceptable representative, and the investigator-designated research professional obtaining the consent.

# Budget & Finance

This trial is funded by the AHSC (Academic Health Science Centers) AFP (Alternate Funding Plan) Innovation Fund. The study drug (Curosurf®) will be provided in-kind by Chiesi Pharmaceuticals S.p.A.

# Publication Plan

Results of the study will be presented at relevant national and international scientific meetings and submitted for publication in a relevant peer-reviewed journal. Approval from the primary responsible party (Dr. Brigitte Lemyre) must be obtained before any information can be used or passed on to a third party.

# References

(1) Seger N, Soll R. Animal derived surfactant extract for treatment of respiratory distress syndrome. Cochrane Database Syst Rev 2009; (2): CD007836.

(2) Schmidt B, Asztalos EV, Roberts RS, Robertson CM, Sauve RS, Whitfield MF. Impact of bronchopulmonary dysplasia, brain injury, and severe retinopathy on the outcome of extremely low-birth-weight infants at 18 months: results from the trial of indomethacin prophylaxis in preterms. JAMA 2003; 289 (9): 1124-9.

(3) Morley CJ, Davis PG, Doyle LW, Brion LP, Hascoet JM, Carlin JB. Nasal CPAP or intubation at birth for very preterm infants. N Engl J Med 2008; 358 (7): 700-8.

(4) Dunn MS, Kaempf J, de KA, de KR, Reilly M, Howard D, et al. Randomized Trial Comparing 3 Approaches to the Initial Respiratory Management of Preterm Neonates. Pediatrics 2011; 128 (5): e1069-e1076.

(5) Finer NN, Carlo WA, Walsh MC, Rich W, Gantz MG, Laptook AR, et al. Early CPAP versus surfactant in extremely preterm infants. N Engl J Med 2010; 362 (21): 1970-9.

(6) Tarawneh A, Kaczmarek J, Bottino MN, Sant'anna GM. Severe airway obstruction during surfactant administration using a standardized protocol: a prospective, observational study. J Perinatol 2011.

(7) Sandri F, Plavka R, Ancora G, Simeoni U, Stranak Z, Martinelli S, et al. Prophylactic or early selective surfactant combined with nCPAP in very preterm infants. Pediatrics 2010; 125 (6): e1402-e1409.

(8) Gharehbaghi MM, Sakha SH, Ghojazadeh M, Firoozi F. Complications among premature neonates treated with beractant and poractant alfa. Indian J Pediatr 2010; 77 (7): 751-4.

(9) Baroutis G, Kaleyias J, Liarou T, Papathoma E, Hatzistamatiou Z, Costalos C. Comparison of three treatment regimens of natural surfactant preparations in neonatal respiratory distress syndrome. Eur J Pediatr 2003; 162 (7-8): 476-80.

(10) Singh N, Hawley KL, Viswanathan K. Efficacy of Porcine Versus Bovine Surfactants for Preterm Newborns With Respiratory Distress Syndrome: Systematic Review and Meta-analysis. Pediatrics 2011; 128 (6): e1588-e1595.

(11) Gopel W, Kribs A, Ziegler A, Laux R, Hoehn T, Wieg C, et al. Avoidance of mechanical ventilation by surfactant treatment of spontaneously breathing preterm infants (AMV): an open-label, randomised, controlled trial. Lancet 2011; 378 (9803): 1627-34.

(12) Dargaville PA, Aiyappan A, Cornelius A, Williams C, De Paoli AG. Preliminary evaluation of a new technique of minimally invasive surfactant therapy. Arch Dis Child Fetal Neonatal Ed 2011; 96 (4): F243-F248.

(13) Peliowski A. A Randomized, blinded, Canadian multi-center trial, to compare a bovine surfactant, bLES (R) (b), with a synthetic Exosurf (E) for the rescue treatment of RDS in premature newborns < or = 1250g. Pediatr Research 43[4]. 1998.
Ref Type: Generic

(14) Lam BC, Ng YK, Wong KY. Randomized trial comparing two natural surfactants (Survanta vs. bLES) for treatment of neonatal respiratory distress syndrome. Pediatr Pulmonol 2005; 39 (1): 64-9.

(15) Fujii AM, Patel SM, Allen R, Doros G, Guo CY, Testa S. Poractant alfa and beractant treatment of very premature infants with respiratory distress syndrome. J Perinatol 2010; 30 (10): 665-70.

(16) Kliegman RM, Walsh MC. Neonatal necrotizing enterocolitis: pathogenesis, classification, and spectrum of illness. Curr Probl Pediatr 1987; 17 (4): 213-88.

(17) Papile LA, Burstein J, Burstein R, Koffler H. Incidence and evolution of subependymal and intraventricular hemorrhage: a study of infants with birth weights less than 1,500 gm. J Pediatr 1978; 92 (4): 529-34.

(18) Pinto-Martin JA, Riolo S, Cnaan A, Holzman C, Susser MW, Paneth N. Cranial ultrasound prediction of disabling and nondisabling cerebral palsy at age two in a low birth weight population. Pediatrics 1995; 95 (2): 249-54.

(19) Screening examination of premature infants for retinopathy of prematurity. Pediatrics 2006; 117 (2): 572-6.

(20) Jobe AH, Bancalari E. Bronchopulmonary dysplasia. Am J Respir Crit Care Med 2001; 163 (7): 1723-9.

| **Timetable of Events** | | | | | | | | | | | |
| --- | --- | --- | --- | --- | --- | --- | --- | --- | --- | --- | --- |
| **Evaluation** | **Baseline** | **30 m.**  **(± 15 min)** | **6 hrs**  **(± 30 min)** | **12 hrs**  **(± 2 hrs)** | **24 hrs**  **(± 2 hrs)** | **36 hrs**  **(± 2 hrs)** | **48 hrs**  **(± 2 hrs)** | **Week 2**  **(±1 week)** | **Week 4**  **(±1 week)** | **Week 8^*^**  **(±1 week)** | **Discharge from hospital**  **(36 wksGA)** |
| Consent ^1^ | X |  |  |  |  |  |  |  |  |  |  |
| Medical/Medication History | X |  |  |  |  |  |  |  |  |  |  |
| Physical Exam ^2^ | X |  |  |  |  |  |  |  |  |  |  |
| Demographics | X |  |  |  |  |  |  |  |  |  |  |
| Randomization | X |  |  |  |  |  |  |  |  |  |  |
| Ventilator parameters^3^ | X | X | X | X | X | X | X |  |  |  |  |
| Con Meds | X |  |  |  |  |  | X | X | X | X | X |
| SAE assessment |  | X | X | X | X | X | X | X | X | X | X |
| Extubation failure assessment |  |  |  |  |  |  |  | X | X | X | X |
| Secondary outcome assessment |  |  |  |  |  |  |  |  |  |  | X |

# Appendices

# Appendix A: Time table of Events

^1^ Consent may be obtained from the mother antenatally or after the birth of the infant.

^2^ Includes weight, Temperature (Axillary), heart rate (apical), respirations.

^3^ Includes Rate, PIP, PEEP, FiO2, Mean, IT, and Tidal volume

**^*^** Data collection at this time point is not done if the infant has already been discharged.

# Appendix B: Extubation Criteria

Infants enrolled in the study will be extubated when they meet all of the following criteria:

1. rate on ventilator ≤40 per minute and
2. mean airway pressure ≤ 10 cm H_2_0 and
3. fi0_2_ ≤ 30%

Individual physicians can extubate patients from higher settings, at their discretion.

# Appendix C: Extubation Failure Criteria

The following are the criteria used to decide to reintubate a baby <30 weeks gestational age after a period of extubation on non-invasive respiratory support or low flow oxygen. Small variances of these parameters are allowed within the protocol (indicated in brackets),

RDS score (validated respiratory distress score) > 8 OR >1 of the following:

1) FiO2 ≥ 40% (±2%) & climbing;

2) pH<7.20(±0.1) and pCO2>60 (±2 ), (persistent & associated with clinical distress) or

3) recurrent apnea (6/6h requiring stimulation or >2 on 2 consecutive hours or 1 requiring bagging).

A baby will be considered as having failed extubation if he/she requires reintubation within 72 hours of extubation.

# Appendix D: Secondary Outcomes

1. Extubation failure (defined as in Appendix C )

2. Duration of first intubation (in hours/days)

3. Total duration of respiratory support (ventilator and nCPAP) and

Total number of days of oxygen requirement

4. Number of doses of surfactant received

5. Adverse events during or after administration of surfactant:

a) Severe airway obstruction, defined as sudden and severe desaturation < 80% with bradycardia (heart rate <100 bpm) with absence of visualization of chest movements with no response to increases in ventilator pressure or bagging for ≥3 minutes and requiring suctioning of surfactant or change of endotracheal tube (ETT)

b) Need for cardiopulmonary resuscitation

c) Pulmonary hemorrhage, defined as fresh blood in the ETT lumen associated with an increase in ventilation or oxygen requirements

d) Pulmonary air leaks (pneumothorax and/or pulmonary interstitial emphysema (PIE) on CXR)

6. Bronchopulmonary dysplasia, defined as oxygen or respiratory support requirement at 36 weeks corrected GA

7. Mortality prior to discharge

# Appendix E: SOP for study drug administration

#

# Appendix F: PRODUCT MONOGRAPH-Curosurf®

#

#

# Appendix G: PRODUCT MONOGRAPH-BLES®

# Appendix H: Informed Consent

**Notes:**
